# International Clinics

**Published:** 1904-01

**Authors:** 


INTERNATIONAL CLINICS. A Quarterly of Clinical Lectures and Especially Prepared Articles of Medicine, Neurology, Surgery, Therapeutics, Obstetrics, Pathology, Pediatrics, Dermatology, Disases of the Eye, Ear, Nose and Throat and Other Topics of Interest to Students and Practitioners. By leading members of the medical profession throughout the world, with regular correspondents in Montreal, London, Paris, Leipsic and Vienna.

Three volumes are before the reviewer, one of the eleventh series and two of the twelfth series. Published by J. B. Lippincott Co. Price, $2.00 each.

